# Altered amygdala volumes and microstructure in focal epilepsy patients with tonic–clonic seizures, ictal, and post‐convulsive central apnea

**DOI:** 10.1111/epi.17804

**Published:** 2023-10-31

**Authors:** Claudia Zeicu, Antoine Legouhy, Catherine A. Scott, Joana F. A. Oliveira, Gavin P. Winston, John S. Duncan, Sjoerd B. Vos, Maria Thom, Samden Lhatoo, Hui Zhang, Ronald M. Harper, Beate Diehl

**Affiliations:** ^1^ Department of Clinical and Experimental Epilepsy, UCL Queen Square Institute of Neurology University College London London UK; ^2^ Centre for Medical Image Computing and Department of Computer Science University College London London UK; ^3^ Department of Clinical Neurophysiology University College London Hospitals NHS Foundation Trust National Hospital for Neurology and Neurosurgery London UK; ^4^ Epilepsy Society MRI Unit Chalfont St Peter UK; ^5^ Department of Medicine, Division of Neurology Queen's University Kingston Ontario Canada; ^6^ Neuroradiological Academic Unit, UCL Queen Square Institute of Neurology University College London London UK; ^7^ Centre for Microscopy, Characterisation, and Analysis The University of Western Australia Nedlands Western Australia Australia; ^8^ Department of Neurology University of Texas Health Sciences Center at Houston Houston Texas USA; ^9^ Brain Research Institute University of California at Los Angeles Los Angeles California USA; ^10^ Department of Neurobiology, David Geffen School of Medicine University of California at Los Angeles Los Angeles California USA

**Keywords:** amygdala, apnea, diffusion MRI, NODDI, SUDEP, tonic–clonic seizures

## Abstract

**Objectives:**

Sudden unexpected death in epilepsy (SUDEP) is a leading cause of death for patients with epilepsy; however, the pathophysiology remains unclear. Focal‐to‐bilateral tonic–clonic seizures (FBTCS) are a major risk factor, and centrally‐mediated respiratory depression may increase the risk further. Here, we determined the volume and microstructure of the amygdala, a key structure that can trigger apnea in people with focal epilepsy, stratified by the presence or absence of FBTCS, ictal central apnea (ICA), and post‐convulsive central apnea (PCCA).

**Methods:**

Seventy‐three patients with focal impaired awareness seizures without FBTC seizures (FBTCneg group) and 30 with FBTCS (FBTCpos group) recorded during video electroencephalography (VEEG) with respiratory monitoring were recruited prospectively during presurgical investigations. We acquired high‐resolution T1‐weighted anatomic and multi‐shell diffusion images, and computed neurite orientation dispersion and density imaging (NODDI) metrics in all patients with epilepsy and 69 healthy controls. Amygdala volumetric and microstructure alterations were compared between three groups: healthy subjects, FBTCneg and FBTCpos groups. The FBTCpos group was further subdivided by the presence of ICA and PCCA, verified by VEEG.

**Results:**

Bilateral amygdala volumes were significantly increased in the FBTCpos cohort compared to healthy controls and the FBTCneg group. Patients with recorded PCCA had the highest increase in bilateral amygdala volume of the FBTCpos cohort.

Amygdala neurite density index (NDI) values were decreased significantly in both the FBTCneg and FBTCpos groups relative to healthy controls, with values in the FBTCpos group being the lowest of the two. The presence of PCCA was associated with significantly lower NDI values vs the non‐apnea FBTCpos group (p = 0.004).

**Significance:**

Individuals with FBTCpos and PCCA show significantly increased amygdala volumes and disrupted architecture bilaterally, with greater changes on the left side. The structural alterations reflected by NODDI and volume differences may be associated with inappropriate cardiorespiratory patterns mediated by the amygdala, particularly after FBTCS. Determination of amygdala volumetric and architectural changes may assist identification of individuals at risk.

**Key points**

- Centrally‐mediated respiratory depression may increase the risk of SUDEP.
- Patients with PCCA had the highest increase in bilateral amygdala volume.
- Neurite density index was significantly lower in PCCA group compared to non‐apnea FBTCpos cohort.
- Recognition of amygdala microstructure alterations may assist in risk stratifying individual patients.

## 1 INTRODUCTION

Sudden unexpected death in epilepsy (SUDEP) is a leading cause of premature death in people with epilepsy; however, the pathophysiology behind the fatal events remains unclear.^1^ The presence of frequent tonic–clonic seizures is a major risk factor.^2^

Several mechanisms have been proposed to precipitate SUDEP, including interictal or postictal hypoxemia triggered by apnea, or profound loss of blood pressure elicited by arrhythmia, or asystole followed by terminal cardiac arrest.^2^, ^3^ The incidence and mechanisms of cardiorespiratory arrests in an epilepsy monitoring unit (MORTEMUS) study^2^ highlighted peri‐ictal and post‐ictal respiratory dysfunction as an initiating abnormality that eventually leads to cardiac arrythmia, asystole, and death. A few studies evaluating the incidence of seizures associated with respiratory dysfunction suggested that post‐convulsive central apnea (PCCA) may be a clinical biomarker of SUDEP.^3^, ^4^

Patients with epilepsy who succumbed to SUDEP, or those who are in the high‐risk category for a fatal outcome, showed significant brain structural alterations in gray matter that serve major roles in maintaining breathing and blood pressure, and in recovery from a failure in those systems.^5^, ^6^, ^7^ The microstructure of those sites, and how alterations in structure might contribute to ictal central apnea (ICA) or PCCA remain to be described.

Temporal lobe epilepsy is the most common form of focal epilepsy, and the hippocampus accounts for the majority of seizure onsets.^8^ The amygdala, a temporal lobe structure with pronounced hippocampal and other temporal lobe projections, often participates in temporal lobe seizures. Amygdala structures receive widespread projections from additional cortical and subcortical sites. A principal concern in seizures involving the medial temporal structures is the pronounced downstream projections to cardiovascular regulatory sites and respiratory timing and drive areas of the brainstem. Normally these amygdala influences trigger breathing and cardiovascular responses to affective stimuli, particularly fear and anxiety, but a role in non‐emotional breathing and cardiovascular control has been recognized.^9^, ^10^, ^11^, ^12^

Stimulation of the human amygdala triggers apnea, sometimes without subject awareness of breathing cessation,^13^, ^14^, ^15^ leading to the hypothesis that amygdala influences may affect respiratory recovery following seizures. Those influences may be enhanced or disrupted if the amygdala is damaged by recurrent seizures and epileptogenesis.

The aim was to assess the volume and microstructure alterations of the amygdala in conjunction with breathing parameters, including ICA and PCCA, across three groups: healthy controls, participants with focal impaired awareness seizures without FBTC seizures (FBTCneg group), and participants with focal‐to‐bilateral tonic–clonic seizures (FBTCpos group). We also examined evidence that inappropriate cardiorespiratory patterns mediated by the amygdala may be used to identify individuals at risk.

## 2 MATERIALS AND METHODS

### 2.1 Study design

Participants were recruited prospectively at the National Hospital for Neurology and Neurosurgery, London, as part of investigation to determine autonomic and imaging biomarkers of SUDEP (Center for SUDEP Research; CSR). All subjects gave written informed consent, and the study was approved by the Research Ethics Committee (19/SW/00071). The MRI data sets were part of the CSR. All enrolled participants with epilepsy had video electroencephalography (VEEG) and respiratory pattern monitoring using respiratory belts and SpO_2_.

All patients had focal epilepsy and were stratified into those FBTCneg and those who were FBTCpos.

In addition, included patients in the FBTCneg group had at least one recorded focal impaired awareness seizure with adequate respiratory monitoring during video telemetry, and FBTCpos subjects had at least one recorded FBTC seizure. All participants underwent a standardized magnetic resonance imaging (MRI) protocol, and all patients had ongoing seizures at the time of the imaging. Historical seizure type data were evaluated for the full cohort of patients irrespective of seizure type. Patients with lesions on MRI, such as focal cortical dysplasia, sclerosis, gliosis, cavernous angioma, or other abnormal brain tissue were excluded. All included cases showed no lesions on MRI examination.

### 2.2 MRI: acquisition and image processing

Images from the study participants were acquired from the same 3 T GE MR750 scanner. Participants underwent high‐resolution (1 mm × 1 mm × 1 mm) three‐dimensional (3D) T1‐weighted anatomic acquisitions and advanced multi‐shell diffusion‐weighted imaging (DWI) optimized for NODDI: 11 b = 0 and diffusion‐weighted images with b = 300, 700, and 2500 with respectively 8, 32, and 64 directions; voxel size: 2 mm × 2 mm × 2 mm.^16^

Amygdala segmentation was performed on the T1‐weighted images using Freesurfer (Version 7.0.0, Martinos Center for Biomedical Imaging),^17^, ^18^ and the operator was blinded to each participant's subgroup.

Diffusion‐weighted images were corrected for tissue magnetic susceptibility‐induced distortion, subject motion, and eddy current‐induced distortions using FSL TOPUP^19^ and FSL EDDY.^20^ The diffusion tensor imaging (DTI) model was fitted through FSL DTIFIT (only using b = 0, 300 and 700) from which we extracted mean diffusivity (MD) and fractional anisotropy (FA) maps. The neurite orientation dispersion and density imaging (NODDI) model was fitted using the NODDI Matlab toolbox^21^ to extract orientation dispersion index (ODI) and the neurite density index (NDI).^22^ Taking advantage of the FWF computed when fitting NODDI, tissue‐weighted mean^23^ was used as region of interest (ROI)–wise statistic to cope with free water contamination. NODDI aims to provide further information regarding tissue‐specific indices such as NDI, which evaluates the density of axons or dendrites, and ODI, which assesses the extent of axons or dendritic projections being incoherently oriented (non‐parallel).^22^

### 2.3 Population

Overall, 154 patients with epilepsy completed the full imaging protocol and had associated respiratory parameters available. After imaging review, 25 patients were removed due to hippocampal sclerosis. A further 11 patients were excluded from the study due to historical seizure type data (patients in the FBTCneg cohort had ongoing FBTC seizures earlier including at the time of imaging). Finally, eight patients were excluded, as they had no recorded seizure activity on VEEG.

A total of 103 patients with epilepsy and 69 healthy controls were eligible for the study. The cohort was initially divided into three groups: healthy controls, FBTCneg cohort (never, or rare historical FBTCS, but not for several years), and FBTCpos cohort with ongoing FBTC seizures. Subsequently, both FBTCneg cohort and the FBTCpos cohort were each divided, depending on the presence or absence of ICA. In a separate analysis, the FBTCpos cohort was subdivided depending on the presence or absence of PCCA. ICA and PCCA presence throughout the cohort was verified by two independent telemetry unit neurophysiologists who examined the EEG and respiratory parameters until the EEG returned to the pre‐ictal state. When respiratory bands or oximeter values were not available, the neurophysiologists reviewed the video telemetry until EEG returned to baseline. All apnea‐related behavioral inferences, such as talking or nursing interactions were excluded. An apnea was defined as one or more missed breaths, as in previous studies.^4^

### 2.4 Statistical methods

Amygdala volume and microstructure differences between groups were assessed using a multivariate analysis of covariance (MANCOVA), controlling for age and sex. The dependent variables were the diffusion metrics (FA, MD, NDI, ODI) and volume values for each individual participant. The null hypothesis (H0) was that the means across groups were equal for each diffusion metric and volume. If the MANCOVA values differed significantly, an analysis of covariance (ANCOVA) was conducted for each diffusion metric or volume individually, controlling for age and sex. Bonferroni correction was used post test to counteract for multiple comparisons with a family‐wise error rate (FWER) of 5%. An independent *t* test with Welch comparison was used for the temporal lobe epilepsy (TLE) and extratemporal lobe epilepsy (ETLE) comparison.

The statistical analysis used IBM SPSS Statistics Data Editor (Version 28.0, IBM Corporation). The statistical figures were designed using Prism 9 (GraphPad).

The data sets used and analyzed during the current study are available from the senior and corresponding authors on request.

## 3 RESULTS

### 3.1 Participant characteristics

The participants’ demographics and epileptogenic zones are summarized in Table 1. A one‐way ANOVA was performed to compare the participants’ ages across the main three cohorts, and it showed that the mean ages between at least two groups differed significantly (F (2,170) = [7.584], *p* = .0001). The mean age between groups differed statistically, as demonstrated by an unpaired *t* test with Welch corrections. NODDI, DTI, and volume statistical data used a statistical analysis that corrects for the age of the cohort.

**TABLE 1:** Demographics and epilepsy characteristics.

Characteristics	FBTCpos cohort (*n* = 30)	FBTCneg cohort (*n* = 73)	Healthy controls (*n* = 69)
Age, mean (SD), years	30.84 ± 6.38	34.58 ± 11.98	40.82 ± 12.95
Sex, *n*			
Male	22	36	25
Female	8	38	43
Duration of epilepsy, mean, years (range)	19.54 (3–39)	18.29 (4–58)	–
Seizure frequency, mean per year (range)	18.5 (1–168)	182.23 (12, 1095)	–
Epileptogenic hemisphere, *n* (%)			
Left hemisphere	15 (50)	26 (35.61)	–
Right hemisphere	7 (23.33)	35 (47.95)	–
Multifocal	5 (16.67)	10 (13.70)	–
Unknown	3 (10)	2 (2.74)	–
Epileptogenic zone, *n* (% of each group)			
Temporal	12 (40.01)	26 (35.61)	–
Temporo‐occipital	0	2 (2.74)	–
Fronto‐temporal	1 (3.33)	3 (4.11)	–
Insula	0	1 (1.37)	–
Frontal	4 (13.33)	13 (17.81)	–
Parieto‐occipital	0	1 (1.37)	–
Parietal	1 (3.33)	0	–
Multifocal	5 (16.67)	10 (13.70)	–
Hemispheric	4 (13.33)	15 (20.55)	–
Unknown	3 (10)	2 (2.74)	–
Seizures recorded on VEEG, *n*	150	451	–
Participants with ICA, *n* (% of each group)	17 (56.67)	36 (49.32)	–
Seizures with ICA, *n* (% of each group)	26 (17.33)	103 (22.84)	–
Duration of ICA, median (range), seconds	12.5 (4–38)	10.5 (4–40)	–
Participants with PCCA, *n* (% of each group)	5 (16.67)	–	–
Seizures with PCCA, *n* (% of each group)	11 (8.53)	–	–
Duration of PCCA, mean (range), seconds	12.13 (5–28)	–	–

Abbreviations: ICA, ictal central apnea; PCCA, post‐convulsive central apnea.

### 3.2 Respiratory data

Data from respiratory bands were available in at least one focal‐impaired awareness seizure in the FBTCneg cohort, and at least one FBTCS in the FBTCpos cohort, overall in 291 of 601 recorded seizures. Pulse oximetry with sufficient quality was recorded in 176 of a total of 601 seizures from 39 patients. Thirty‐six patients with ictal central apnea had ictal oxygen saturation (SpO_2_) available for analysis. Baseline SaO_2_ (M = 95.43, SD = 1.72) and ICA SaO_2_ (M = 88.20, SD = 13.90) differed significantly differed; paired‐sample *t* test, *t*(86) = 3.424, *p* = .0009).

### 3.3 Amygdala volume

First, we evaluated the left and right amygdala volumes, stratified into three groups: healthy controls, FBTCneg cohort, and FBTCpos cohort.

**FIGURE 1:**
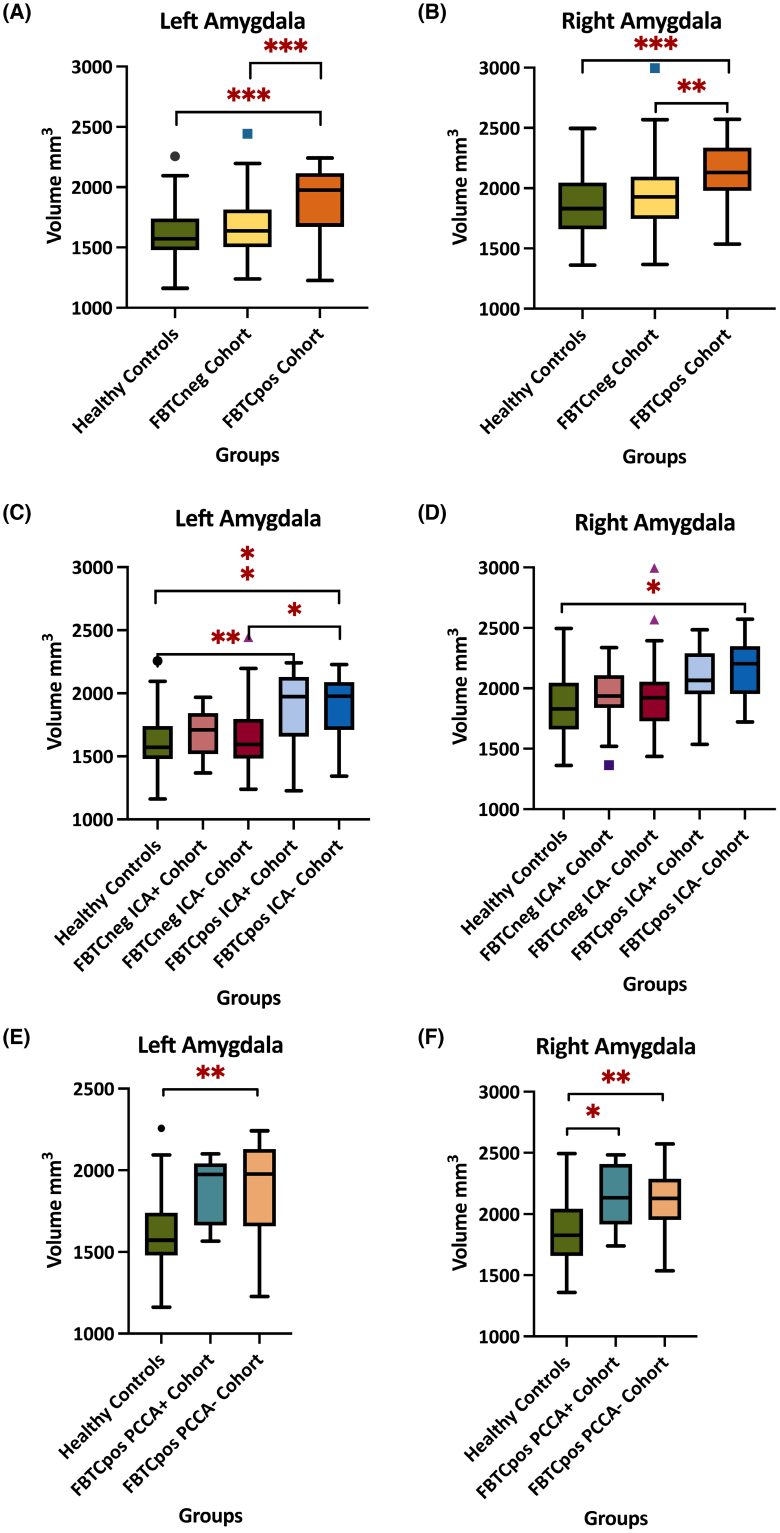
Amygdala Volume Tukey Box Plots. Line is median, and the whiskers are min and max values with some dots as the extreme outliers. FBTC, focal‐to‐bilateral tonic–clonic; ICA, ictal central apnea; PCCA, post‐convulsive central apnea (* < 0.05, ** < 0.01, *** < 0.001). (A) left amygdala volume mm^3^ in 172 participants subdivided into healthy controls, FBTCneg cohort, and FBTC seizures. (B) right amygdala volume mm^3^ in 172 participants subdivided into healthy controls, FBTCneg cohort, and FBTCpos. (C) left amygdala volume groups further divided in conjunction with the presence or absence of ICA. (D) right amygdala volume groups further divided in conjunction with the presence or absence of ICA. (E) left amygdala volume groups further divided in conjunction with the presence or absence of PCCA. (F) Left amygdala volume groups further divided in conjunction with the presence or absence of PCCA.

The pairwise comparison post hoc test with Bonferroni correction showed that the left amygdala volumes were increased significantly in the FBTCpos cohort compared to healthy controls (*p* < .001) and the FBTCneg cohort (*p* < .001) (Figure 1A). Right amygdala volumes in the FBTCpos group showed a significant volume increase compared to healthy controls (*p* < .001) and the FBTCneg group (*p* = .008) (Figure 1B). Graphical representations of the mean volume distribution of the left and right amygdala are in Figure 1, whereas the detailed results are outlined in Tables S1 and S2.

Second, we compared the amygdala volumes between epilepsy groups with and without FBTCs, ipsilateral and contralateral to the epileptogenic zone in TLE and ETLE. In all (TLE and ETLE combined), people with FBTC seizures had larger amygdala volumes both ipsilateral and contralateral to the epileptogenic zone.

In TLE, larger volumes were found in people with FBTC seizures contralateral to the epileptogenic zone (Table S3).

Third, amygdala volumes were assessed relative to breathing parameters, and the groups were subdivided according to the presence or absence of ICA. Left amygdala volumes were lowest in the healthy controls, followed by the FBTCneg without ICA (Figure 1C), but did not differ significantly from healthy controls (*p* = 1.000). The FBTCpos cohort without ICA showed a significant volume increase compared to controls (*p* = .006) and the FBTCneg cohort (*p* = .010). The right amygdala volumes showed a similar distribution, with the FBTCpos cohort without ICA showing a significant volume increase compared to healthy controls (*p* = .013) (Figure 1D). The FBTCpos cohort with or without ICA involvement during seizures showed an overall mean volume increase when compared to controls.

The left and right amygdala volumes were examined in association with the PCCA identified in the FBTCpos cohort (Figure 1E,F). Comparisons between healthy controls and FBTCpos participants were subdivided according to the presence or absence of PCCA. In the left amygdala, both the PCCA– FBTCpos cohort and PCCA+ FBTCS group showed increased volumes compared to healthy controls (Figure 1E,F), with only statistically significant volume differences found between healthy controls and the FBTCpos group without PCCA (*p* = .002). Of note, in the right amygdala, both FBTCpos groups showed statistically significant volume increases compared to healthy controls.

### 3.4 DTI metrics

DTI showed similar MD and FA values in the left and right amygdala between FBTCneg, FBTCpos cohorts, and healthy controls.

Once the groups were subdivided according to presence of ICA, no statistically significant differences between groups in the left amygdala emerged. However, the right amygdala MD trend was significantly higher in the FBTCpos ICA‐seizure cohort compared to healthy controls (*p* = .014) when adjusted for multiple comparisons using the Bonferroni correction. A similar pattern was found between the FBTCneg ICA‐cohort and controls (*p* = .044).

Split by the presence or absence of PCCA, the ANCOVA showed significant differences between groups in the left amygdala in both the MD and FA metrics. The FBTCpos PCCA+ seizure cohort showed a significantly higher FA than in the FBTCpos group (*p* = .037). There was no statistically significant difference between the FBTCpos PCCA+ seizure cohort and healthy controls; however, the FBTCpos PCCA– seizure cohort had a significantly lower FA when compared to FBTCpos PCCA+ group (*p* = .037). Significantly lower FA values were found in the FBTCpos PCCA– seizure cohort when compared to healthy controls (*p* = .008). No statistically significant differences emerged between FBTCpos PCCA+ group and healthy controls in the MD metrics (*p* = 1.000); however, statistically higher MD values appeared in the left amygdala FBTCpos PCCA– group compared to controls. In the right amygdala, a similar significant increase in MD was found between the FBTCpos PCCA– group and healthy controls (*p* = .022).

### 3.5 NODDI metrics

The left and right amygdala NDI values were significantly lower in both the FBTCneg and FBTCpos groups than in healthy controls (*p* < .001), with the FBTCpos group being the lowest of the two (Figure 1A,B).

**FIGURE 2:**
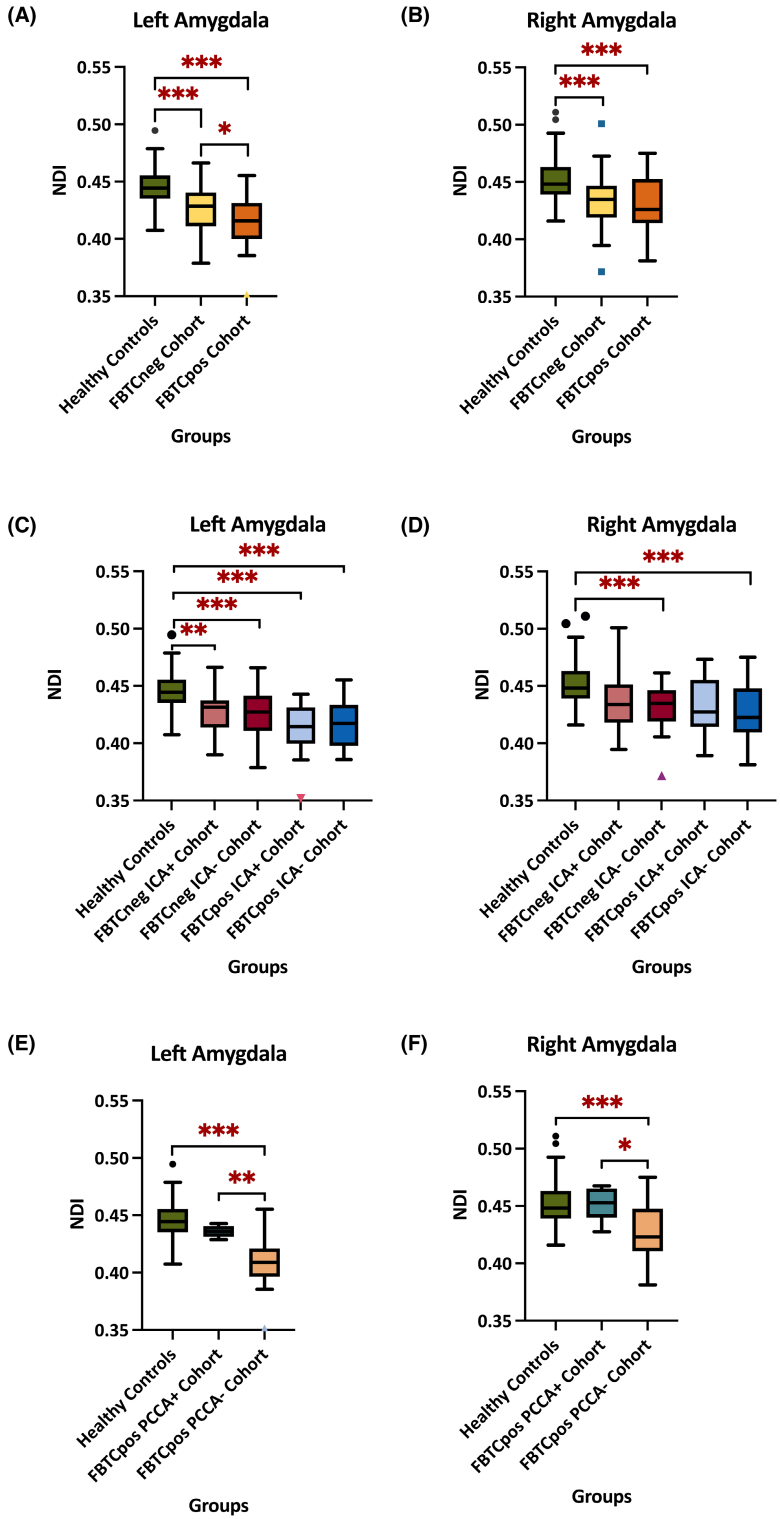
NDI Tukey Box Plot. Line is median, and the whiskers are min and max values with some dots as the extreme outliers. Abbreviations: NDI, neurite dispersion index; FBTC, focal to bilateral tonic–clonic; ICA, ictal central apnea; PCCA, post‐convulsive central apnea (* < 0.05, ** < 0.01, *** < 0.001). (A) left amygdala NDI in 172 participants subdivided into healthy controls, FBTCneg cohort, and FBTCpos. (B) right amygdala NDI in 172 participants subdivided into healthy controls, FBTCneg cohort, and FBTCpos. (C) Left amygdala NDI groups further divided in conjunction with the presence or absence of ICA. (D) Right amygdala NDI groups further divided in conjunction with the presence or absence of ICA. (E) Left amygdala NDI groups further divided in conjunction with the presence or absence of PCCA. (F) left amygdala volume groups further divided in conjunction with the presence or absence of PCCA.

A one‐way ANCOVA revealed that statistically significant differences in NDI appeared between the four groups and healthy controls once the patient cohort was divided according to ICA (Figure 1C,D). The most pronounced declines were found between healthy controls and the FBTCneg ICA– Seizure Cohort (*p* < .001), controls vs the FBTCpos ICA+ Seizure Cohort (*p* < .001), and controls vs the FBTCpos ICA– Seizure Cohort (*p* < .001). There were no significant differences between the FBTCneg ICA+ Seizure Cohort and the FBTCpos ICA+ Seizure Cohort or the FBTCneg ICA– Seizure Cohort vs the FBTCpos ICA– Seizure Cohort. A significant decline was found in NDI in the PCCA subgroup vs the FBTCpos group (left amygdala: *p* = .004; right amygdala: *p* = .042) (Figure 2E,F).

The ODI was decreased in both FBTCneg and FBTCpos groups relative to healthy controls, with the only statistically significant decline found in the left amygdala between the FBTCneg seizure cohort and healthy controls (*p* = .013). Of note, patients with recorded PCCA had the greatest decrease in bilateral amygdala ODI values of the population.

Comparing epilepsy groups only, NDI values were lower in the FBTCpos TLE group ipsilateral to the epileptogenic zone (Table S3).

## 4 DISCUSSION

### 4.1 Overview

Nearly a quarter of seizures recorded in this study were accompanied by impaired central breathing control associated with seizure activity, with ICA found in more than half of all cases, a prevalence similar to that of previous reports.^24^, ^25^

The amygdala volume findings showed a strong association between the volume of gray matter changes and the presence or absence of FBTCS in conjunction with ICA and PCCA, an association not previously reported. A key finding was that patients in the FBTCpos PCCA group experienced the highest volume gain relative to healthy controls.

Amygdala ODI and NDI were decreased in the FBTCneg and FBTCpos groups relative to healthy controls, most prominently in the FBTCpos group.

Detailed demographics of the participants revealed a statistically significant difference in mean age across the epilepsy groups and the healthy controls. Female‐to‐male ratios were comparable between the FBTCneg cohort and healthy controls, with the FBTCpos groups comprising a majority of males. However, because the data analyses were controlled for age and sex, these factors should not contribute to major result differences.

The majority of the FBTCneg epilepsy patient cohort had seizure onsets in the temporal lobes; however, a large proportion of the patients with epilepsy had an indeterminate onset, which could be described only as hemispheric or multi‐focal. Despite the unspecified onset of some patients, the amygdala is likely to have become involved during ictal spread from its substantial reciprocal projections with other temporal and extratemporal structures. Seizure activity affecting the amygdala can cause ICA,^15^, ^26^ with direct electrical stimulation inducing apnea.^13^, ^15^ The potential for amygdala involvement via ictal spread is one consideration in understanding the etiology of processes that may be involved in SUDEP.

### 4.2 Amygdala volume and breathing disturbances

Significant volume increases in the amygdala appeared in epilepsy patients, with subjects having PCCA showing the most extensive increase in volume bilaterally; whereas patients with FBTCneg seizures had the lowest volume increases. Amygdala volumes were increased in patients with FBTCpos compared to the FBTCneg group, irrespective of whether the structure was ipsilateral or contralateral to the epileptogenic zone.

The volume alterations are relevant, because of the formation's marked role in integrating signals provided from a wide range of afferent receptors and projecting signals to the lower brain. The central nucleus of the amygdala, for example, receives a wide range of inputs and then projects to the bed nucleus of the stria terminalis, periaqueductal gray, and parabrachial pons,^27^ and can influence both overall drive and timing of breathing^28^; we showed elsewhere^5^ that these structures are severely affected in patients who are at high risk or who succumbed to SUDEP.

Gray matter volume changes also appear within the hippocampus in SUDEP cases compared to low‐risk participants and healthy controls.^5^, ^7^ Although the pathological changes related to the volume increases have yet to be described, a variety of processes may underlie the changes, including gliosis,^29^ inflammation causing neuronal architecture disruption,^30^ or excitotoxic injury.^31^, ^32^ The neural processes accompanying the altered volumes may directly influence the amygdala's control on both respiratory action and cardiovascular instability as shown here and elsewhere.^33^ Determining the nature of gray matter microstructure changes from tissue samples following surgical resections may further understanding of volume alterations in different epilepsy cohorts.

Amygdala and temporal lobe volumes apparently normalize in patients with epilepsy who respond favorably to antiseizure medications (ASM), compared to initial brain scans.^34^ Volume measurements thus may provide insights into the high‐risk population for SUDEP, and also may provide valuable information in predicting overall seizure freedom.

### 4.3 Amygdala microstructure and breathing disturbances

The processes that may underpin the amygdala volume alterations may also be better understood using imaging techniques that can detect neuronal architecture disruption^35^ and vascular changes.^36^

DTI showed decreased FA in the left amygdala, whereas those FA values were increased on the right, a finding that was more pronounced for the FBTCpos cohort with post‐convulsive central apnea. A decline in FA may be caused by Wallerian degeneration,^37^ cerebrospinal fluid (CSF) contamination, or a change in brain tissue organization as a compensatory mechanism.^38^ The FA does not distinguish between the intra‐axonal or extra‐axonal compartment, which may be modulated by fiber density, CSF volume effects, or orientational dispersion. NODDI can estimate the neuronal architecture of dendrites and axons (NDI, ODI) as well as quantify the extend of CSF contamination within a voxel. Alterations in NODDI may represent changes in the projection fibers or dendritic density. Of note, FA may not be able to reliably distinguish between dendritic projections and unmyelinated axons.^39^

Using NODDI, we were able to disentangle various factors that may cause an FA reduction noted above.^37^, ^38^ We demonstrated additional microstructure differences across the cohorts in NDI and ODI. The lowest ODI appeared in the FBTCneg cohort, an outcome that may be correlated with a reduction in orientation dispersion of the gray matter, which may result from reduced dendritic projections.^22^ The application of NODDI in clinical research has been validated in other conditions,^40^ showing lower ODI values in multiple sclerosis demyelinating spinal cord histologically^41^ and in vivo.^42^

The ODI results are also associated with decreased NDI, which translates to a reduction in neurite density volume, particularly found bilaterally in the FBTCpos group. A similar finding was noted in patients with FBTCneg. A low NDI may represent severe loss of dendritic and axonal projections, which in turn, can also interfere with ODI values, as the orientation dispersion is correlated with the tissue sampled.^42^ NODDI offers an excellent opportunity to further understand the neurite architecture in epilepsy without solely relying on pathological studies.

Dendritic projection loss and reduction in orientation dispersion may partially explain some of the ictal and peri‐ictal dysfunction. The slight asymmetry in values, in both volume and microstructure, between the left and right amygdala may pose challenges for autonomic and respiratory control. However, asymmetric influences from the substantial reciprocal projections of external cortical and other limbic sites on the amygdala must be considered. Especially prominent are those of the insular cortex, with projections to the amygdala central nucleus among other nuclei; these influences are lateralized and are not trivial in function. The vestibular system exerts substantial blood pressure control, and its cortical representation lies principally in the posterior insula.^43^ Stimulation of the right insula leads to hypersympathetic activation,^44^ whereas parasympathetic upregulation is largely influenced by the left insula.^45^ The autonomic effects are not confined to blood pressure; transient elevation of blood pressure reflex leads to apnea.^46^

The right amygdala gray matter volume increase may exaggerate sympathetic activation, as previous studies observed direct sympathetic upregulation when stimulation is applied to the right insula.^47^ However, as opposed to the insula, in the amygdala, the cardiorespiratory consequences do not appear to show laterality.^13^

The asymmetry noted in the amygdala volume and microstructure may also result from differential input from other‐than‐cortical sites, such as the thalamus, parabrachial nucleus (PBN), periaqueductal gray (PAG), and the nucleus of the solitary tract (NTS).^48^ The asymmetrical input may underlie some of the observed differences in laterality of function.^49^ Although both the left and right amygdala are activated to fear responses, the right amygdala appears to play a role in memory retention,^50^ and the right amygdala is more involved in nociception signaling than the left. Furthermore, an fMRI study examining sex influences on amygdala function revealed more involvement of the left amygdala in arousing memory consolidation in women over men.^51^ Two impressions arise from these findings. First, the differential laterality of function may derive from separate inputs to the left vs right amygdala, with the known laterality characteristics of the left vs right insula likely exerting a primary role. Those influences may have the potential to separately modify the extent of injury in an asymmetric fashion to the amygdala, depending on the origin of the influences. Second, the impact of damage to the left or right amygdala may be expressed differently in behavior or physiological action, depending on the laterality of injury.

The amygdala exerts profound influences on the cardiovascular system through projections to structures that regulate blood pressure.^52^, ^53^ The insula/amygdala control of the baroreflex adjusts heart rate with blood pressure, thus determining cardiac output in response to stressful stimuli.^53^, ^54^, ^55^ The amygdala influences are mediated through multiple brainstem structures, including the NTS and parabrachial pons, and both sympathetic and parasympathetic systems are targets. Projections from the central nucleus of the amygdala to the periaqueductal gray (PAG) and parabrachial pons influence both respiratory drive and patterning, and also modify cardiovascular action.^27^ The amygdala can influence triggering/not triggering apnea; pulse stimulation of the central nucleus that projects to the parabrachial pons can elicit inspiratory efforts,^28^ and through the projections to the periaqueductal gray, support breathing. Those influences place the amygdala volume and microstructure alterations in an environment to influence vital factors that may contribute to increasing the risk of sudden death in epilepsy. Further studies are required to correlate the changes observed in the amygdala to individual nuclei, particularly the central nucleus, but also subnuclei that influence the central nucleus and can offer more insights concerning cardiac and respiratory regulation.

### 4.4 Limitations

The number of participants satisfying criteria for FBTCpos associated with post‐convulsive central apnea was very small. Because pulse oximetry and respiratory belt signals were not always of adequate quality for many patients undertaking VEEG monitoring, the true prevalence of inappropriate breathing may be higher. The division into ICA and PCCA groups was limited to the seizures recorded in VEEG. Future studies examining seizures and breathing disturbances would likely need to expand the sample size via multi‐center collaborations to ensure that the significant associations in the data are reproducible in a larger cohort.

In conclusion, increased bilateral amygdala volumes, accompanied by a decline in ODI and NDI in patients with epilepsy, particularly the FBTCpos group, may reflect processes having a direct effect on cardiac and breathing patterns, which may increase the risk of SUDEP. The volume and microstructure changes may be mediated by multiple mechanisms, including local inflammation leading to dendritic projections loss or gliosis, or excitotoxicity elicited by excessive external influences on the amygdala. Recognition of amygdala microstructure alterations may assist in identifying individuals with epilepsy who are at a higher risk for SUDEP.

## AUTHOR CONTRIBUTIONS

**Claudia Zeicu:** formal analysis, visualization, writing – original draft. **Antoine Legouhy**: data curation, resources, writing – review and editing. **Catherine A. Scott**: data curation, resources. **Joana F. A. Oliveira**: data curation, resources. **Gavin Winston**: writing – review and editing. **John S. Duncan**: writing – review and editing. **Sjoerd B. Vos:** resources, conceptualization (supporting), writing – review and editing. **Maria Thom**: writing – review and editing. **Samden Lhatoo**: funding acquisition, writing – review and editing. **Hui Zhang**: writing – review and editing. **Ronald M. Harper**: funding acquisition, conceptualization (supporting), writing – review and editing. **Beate Diehl**: funding acquisition, conceptualization (lead), supervisor, writing – review and editing.

## CONFLICT OF INTEREST STATEMENT

None of the authors has any conflict of interest to disclose. We confirm that we have read the Journal's position on issues involved in ethical publication and affirm that this report is consistent with those guidelines.

## Supporting information


Table S1–S3.
