# Supplementary material for: Altered amygdala volumes and microstructure in focal epilepsy patients with tonic–clonic seizures, ictal, and post‐convulsive central apnea
Source: Epilepsia. 2023 Oct 31;64(12):3307–18. doi: 10.1111/epi.17804 (PMC10952501; doi:10.1111/epi.17804)
Supplement: Supplementary file 1 — Table S1–S3. [file EPI-64-3307-s001.docx]

**Supplementary**:

**Supplementary Table 1. Left amygdala brain volume, DTI, and NODDI results with corrected mean and SD for age. The age corrected for the initial groups (Healthy Controls, Focal impaired awareness without FBTC Cohort and FBTCS Seizure Cohort) as well as the ICA split was equal to 36.47196 while the age corrected for the PCCA split was 37.7971. ANCOVA p-values, corrected for age. Bonferroni correction was used post-test.**

| Left Amygdala | | | | | | | | | | | |
| --- | --- | --- | --- | --- | --- | --- | --- | --- | --- | --- | --- |
| Group | n | Volume | | MD (µm^2^ms) | | FA | | ODI | | NDI |  |
|  |  | Mean | SD | Mean | SD | Mean | SD | Mean | SD | Mean | SD |
| Healthy Controls | 69 | 1641.43 | 27.87 | 8.532 × 10^-4^ | 4.1 × 10^-6^ | 0.239 | 0.002 | 0.421 | 0.003 | 0.444 | 0.002 |
| FBTCneg Cohort | 73 | 1656.12 | 25.29 | 8.628 × 10^-4^ | 3.7 × 10^-6^ | 0.237 | 0.002 | 0.409 | 0.003 | 0.427 | 0.002 |
| FBTCpos Cohort | 30 | 1872.34 | 45.28 | 8.694 × 10^-4^ | 6.6 × 10^-6^ | 0.231 | 0.004 | 0.414 | 0.005 | 0.415 | 0.004 |
|  |  | p-value |  | p-value |  | p-value |  | p-value |  | p-value |  |
| Healthy Controls *vs* FBTCneg Cohort |  | 1.000 |  | 0.254 |  | 1.000 |  | **0.013** |  | **<0.001** |  |
| Healthy Controls *vs* FBTCpos Cohort |  | **<0.001** |  | 1.000 |  | 0.170 |  | 0.594 |  | **<0.001** |  |
| FBTCneg Cohort *vs* FBTCpos Cohort |  | **<0.001** |  | 1.000 |  | 0.354 |  | 1.000 |  | **0.019** |  |
| Left Amygdala split by ICA | | | | | | | | | | | |
| Group | n | Volume | | MD (µm^2^ms) | | FA | | ODI | | NDI | |
|  |  | Mean | SD | Mean | SD | Mean | SD | Mean | SD | Mean | SD |
| Healthy Controls | 69 | 1641.43 | 27.87 | 8.532 × 10^-4^ | 4.1 × 10^-6^ | 0.239 | 0.002 | 0.421 | 0.003 | 0.444 | 0.002 |
| FBTCneg ICA+ Seizure Cohort | 36 | 1664.49 | 43.92 | 8.619 × 10^-4^ | 6.3 × 10^-6^ | 0.235 | 0.003 | 0.415 | 0.005 | 0.428 | 0.004 |
| FBTCneg ICA- Seizure Cohort | 37 | 1651.06 | 31.83 | 8.653 × 10^-4^ | 4.5 × 10^-6^ | 0.237 | 0.003 | 0.406 | 0.003 | 0.426 | 0.003 |
| FBTCpos ICA+  Seizure Cohort | 17 | 1853.72 | 63.37 | 8.645 × 10^-4^ | 9.0 × 10^-6^ | 0.235 | 0.005 | 0.411 | 0.007 | 0.421 | 0.005 |
| FBTCpos ICA-  Seizure Cohort | 13 | 1895.83 | 65.91 | 8.730 × 10^-4^ | 9.4 × 10^-6^ | 0.226 | 0.005 | 0.416 | 0.007 | 0.412 | 0.005 |
|  |  | p-value |  | p-value |  | p-value |  | p-value |  | p-value |  |
| Healthy Controls *vs* FBTCneg ICA+ Seizure Cohort |  | 1.000 |  | 1.000 |  | 1.000 |  | 1.000 |  | **0.002** |  |
| Healthy Controls *vs* FBTCneg ICA- Seizure Cohort |  | 1.000 |  | 0.475 |  | 1.000 |  | **0.009** |  | **<0.001** |  |
| Healthy Controls *vs* FBTCpos ICA+ Seizure Cohort |  | **0.030** |  | 1.000 |  | 1.000 |  | 1.000 |  | **<0.001** |  |
| Healthy Controls *vs* FBTCpos ICA- Seizure Cohort |  | **0.006** |  | 0.551 |  | 0.289 |  | 1.000 |  | **<0.001** |  |
| FBTCneg ICA+ Seizure Cohort *vs* FBTCpos ICA+ Seizure Cohort |  | 0.140 |  | 1.000 |  | 1.000 |  | 1.000 |  | 1.000 |  |
| FBTCneg ICA- Seizure Cohort *vs* FBTCpos ICA- Seizure Cohort |  | **0.010** |  | 1.000 |  | 0.639 |  | 1.000 |  | 0.155 |  |
| Left Amygdala split by PCCA | | | | | | | | | | | |
| Group | n | Volume | | MD (µm^2^ms) | | FA | | ODI | | NDI | |
|  |  | Mean | SD | Mean | SD | Mean | SD | Mean | SD | Mean | SD |
| Healthy Controls | 69 | 1639.04 | 29.75 | 8.524 × 10^-4^ | 3.8 × 10^-6^ | 0.239 | 0.002 | 0.422 | 0.003 | 0.445 | 0.002 |
| FBTCpos PCCA- Seizure Cohort | 25 | 1862.27 | 53.02 | 8.765 × 10^-4^ | 7.1 × 10^-6^ | 0.226 | 0.004 | 0.415 | 0.005 | 0.409 | 0.004 |
| FBTCpos PCCA+ Seizure Cohort | 5 | 1932.36 | 131.61 | 8.403 × 10^-4^ | 1.36 × 10^-5^ | 0.246 | 0.007 | 0.410 | 0.009 | 0.438 | 0.008 |
|  |  | p-value |  | p-value |  | p-value |  | p-value |  | p-value |  |
| Healthy Controls *vs* FBTCpos PCCA- Seizure Cohort |  | **0.002** |  | **0.013** |  | **0.008** |  | 0.728 |  | **<0.001** |  |
| Healthy Controls *vs* FBTCpos PCCA+ Seizure Cohort |  | 0.101 |  | 1.000 |  | 1.000 |  | 0.674 |  | 1.000 |  |
| FBTCpos PCCA- Seizure Cohort *vs* FBTCpos PCCA+ Seizure Cohort |  | 1.000 |  | 0.057 |  | **0.037** |  | 1.000 |  | **0.004** |  |

Abbreviations: ICA, ictal central apnea; PCCA, post-convulsive central apnea; MD, mean diffusivity; FA, fractional anisotropy; ODI, orientation dispersion index; NDI, neurite density index.

**Supplementary Table 2. Right amygdala brain volume, DTI, and NODDI results with corrected mean and SD for age. The age corrected for the initial groups (Healthy Controls,** Focal impaired awareness without FBTC **Seizure Cohort and FBTCS Seizure Cohort), as well as the ICA split was equal to 36.47196, while the age corrected for the PCCA split was 37.7971. ANCOVA p-values, corrected for age. Bonferroni correction was used post-test.**

| Right Amygdala | | | | | | | | | | | |
| --- | --- | --- | --- | --- | --- | --- | --- | --- | --- | --- | --- |
| Group | n | Volume | | MD (µm^2^ms) | | FA | | ODI | | NDI | |
|  |  | Mean | SD | Mean | SD | Mean | SD | Mean | SD | Mean | SD |
| Healthy Controls | 69 | 1890.89 | 29.96 | 8.235 × 10^-4^ | 5.7 × 10^-6^ | 0.249 | 0.003 | 0.425 | 0.003 | 0.453 | 0.004 |
| FBTCneg Cohort | 73 | 1933.71 | 26.91 | 8.408 × 10^-4^ | 5.1 × 10^-6^ | 0.244 | 0.003 | 0.416 | 0.003 | 0.432 | 0.003 |
| FBTCpos Cohort | 30 | 2101.77 | 48.17 | 8.493 × 10^-4^ | 9.2 × 10^-6^ | 0.240 | 0.005 | 0.417 | 0.005 | 0.424 | 0.006 |
|  |  | p-value |  | p-value |  | p-value |  | p-value |  | p-value |  |
| Healthy Controls *vs* FBTCneg Cohort |  | 0.877 |  | 0.080 |  | 0.612 |  | 0.122 |  | **<0.001** |  |
| Healthy Controls *vs* FBTCpos Cohort |  | **<0.001** |  | 0.060 |  | 0.299 |  | 0.506 |  | **<0.001** |  |
| FBTCneg Cohort *vs* FBTCpos Cohort |  | **0.008** |  | 1.000 |  | 1.000 |  | 1.000 |  | 0.672 |  |
| Right Amygdala split by ICA | | | | | | | | | | | |
| Group | n | Volume | | MD (µm^2^ms) | | FA | | ODI | | NDI | |
|  |  | Mean | SD | Mean | SD | Mean | SD | Mean | SD | Mean | SD |
| Healthy Controls | 69 | 1890.89 | 29.96 | 8.235 × 10^-4^ | 5.7 × 10^-6^ | 0.249 | 0.003 | 0.425 | 0.003 | 0.453 | 0.003 |
| FBTCneg ICA+ Seizure Cohort | 36 | 1908.1 | 46.38 | 8.492 × 10^-4^ | 2.63 × 10^-5^ | 0.248 | 0.004 | 0.420 | 0.005 | 0.435 | 0.005 |
| FBTCneg ICA- Seizure Cohort | 37 | 1954.13 | 33.62 | 8.474 × 10^-4^ | 3.65 × 10^-5^ | 0.241 | 0.003 | 0.415 | 0.004 | 0.429 | 0.004 |
| FBTCpos ICA+  Seizure Cohort | 17 | 2069.66 | 66.93 | 8.309 × 10^-4^ | 3.14 × 10^-5^ | 0.245 | 0.006 | 0.418 | 0.007 | 0.433 | 0.008 |
| FBTCpos ICA-  Seizure Cohort | 13 | 2139.57 | 69.61 | 8.727 × 10^-4^ | 9.60 × 10^-5^ | 0.235 | 0.006 | 0.412 | 0.007 | 0.313 | 0.008 |
|  |  | p-value |  | p-value |  | p-value |  | p-value |  | p-value |  |
| Healthy Controls *vs* FBTCneg ICA- Seizure Cohort |  | 1.000 |  | **0.044** |  | 0.603 |  | 0.274 |  | **<0.001** |  |
| Healthy Controls *vs* FBTCneg ICA+ Seizure Cohort |  | 1.000 |  | 1.000 |  | 1.000 |  | 1.000 |  | 0.071 |  |
| Healthy Controls *vs* FBTCpos ICA+ Seizure Cohort |  | 0.172 |  | 1.000 |  | 1.000 |  | 1.000 |  | 0.225 |  |
| Healthy Controls *vs* FBTCpos ICA- Seizure Cohort |  | **0.013** |  | 0.156 |  | 0.417 |  | 1.000 |  | **<0.001** |  |
| FBTCneg ICA+ Seizure Cohort *vs* FBTCpos ICA+  Seizure Cohort |  | 0.474 |  | 1.000 |  | 1.000 |  | 1.000 |  | 1.000 |  |
| FBTCneg ICA- Seizure Cohort *vs* FBTCpos ICA-  Seizure Cohort |  | 0.175 |  | 1.000 |  | 1.000 |  | 1.000 |  | 0.720 |  |
| Right Amygdala split by PCCA | | | | | | | | | | | |
| Group | n | Volume | | MD (µm^2^ms) | | FA | | ODI | | NDI | |
|  |  | Mean | SD | Mean | SD | Mean |  | Mean | SD | Mean | SD |
| Healthy Controls | 69 | 1890.50 | 29.07 | 8.266 × 10^-4^ | 2.93 × 10^-5^ | 0.250 | 0.003 | 0.425 | 0.003 | 0.453 | 0.003 |
| FBTCpos PCCA- Seizure Cohort | 25 | 2074.63 | 51.81 | 8.556 × 10^-4^ | 7.32 × 10^-5^ | 0.236 | 0.005 | 0.418 | 0.005 | 0.418 | 0.006 |
| FBTCpos PCCA+ Seizure Cohort | 5 | 2253.74 | 128.62 | 8.161 × 10^-4^ | 3.15 × 10^-5^ | 0.254 | 0.009 | 0.419 | 0.10 | 0.452 | 0.012 |
|  |  | p-value |  | p-value |  | p-value |  | p-value |  | p-value |  |
| Healthy Controls *vs* FBTCpos PCCA- Seizure Cohort |  | **0.009** |  | **0.022** |  | 0.059 |  | 0.844 |  | **<0.001** |  |
| Healthy Controls *vs* FBTCpos PCCA+ Seizure Cohort |  | **0.023** |  | 1.000 |  | 1.000 |  | 1.000 |  | 1.000 |  |
| FBTCS PCCA- Seizure Cohort *vs* FBTCpos PCCA+ Seizure Cohort |  | 0.579 |  | 0.237 |  | 0.280 |  | 1.000 |  | **0.042** |  |

Abbreviations: ICA, ictal central apnea; PCCA, post-convulsive central apnea; MD, mean diffusivity; FA, fractional anisotropy; ODI, orientation dispersion index; NDI, neurite density index.

**Supplementary Table 3. Ipsilateral and contralateral amygdala brain volume, DTI, and NODDI results. Independent t-test (with Welch corrections) p-values.**

| **Ipsilateral to the Epileptogenic zone** | | | | | | | | | | | |
| --- | --- | --- | --- | --- | --- | --- | --- | --- | --- | --- | --- |
| Group | n | Volume | | MD (µm^2^ms) | | FA | | ODI | | NDI | |
|  |  | Mean | SD | Mean | SD | Mean | SD | Mean | SD | Mean | SD |
| FBTCneg TLE | 26 | 1790.89 | 306.32 | 0.001 | 0.00 | 0.238 | 0.026 | 0.409 | .035 | 0.418 | 0.418 |
| FBTCpos TLE | 12 | 1894.26 | 340.10 | 0.001 | 0.00 | 0.229 | 0.017 | 0.406 | 0.036 | 0.396 | 0.053 |
| FBTCneg TLE and extra-TLE | 61 | 1820.15 | 282.85 | 0.001 | 0.00 | 0.241 | 0.023 | 0.410 | 0.031 | 0.427 | 0.027 |
| FBTCpos TLE and extra-TLE | 22 | 1977.48 | 278.94 | 0.001 | 0.00 | 0.232 | 0.015 | 0.407 | .030 | 0.404 | .041 |
|  |  | p-value |  | p-value |  | p-value |  | p-value |  | p-value |  |
| FBTCneg TLE *vs* FBTCpos TLE |  | 0.382 |  | 0.433 |  | 0.259 |  | 0.827 |  | 0.215 |  |
| FBTCneg TLE and extra-TLE *vs* FBTCpos and extra-TLE |  | **0.029** |  | 0.120 |  | 0.056 |  | 0.762 |  | **0.021** |  |
| **Contralateral to the epileptogenic zone** | | | | | | | | | | | |
| Group | n | Volume | | MD (µm^2^ms) | | FA | | ODI | | NDI | |
|  |  | Mean | SD | Mean | SD | Mean | SD | Mean | SD | Mean | SD |
| FBTCneg TLE | 26 | 1688.54 | 254.75 | 0.001 | 0.00 | 0.238 | 0.019 | 0.416 | 0.028 | 0.432 | 0.016 |
| FBTCpos TLE | 12 | 2002.78 | 365.05 | 0.001 | 0.00 | 0.241 | 0.020 | 0.428 | 0.014 | 0.440 | 0.024 |
| FBTCneg TLE and extra-TLE | 61 | 1726.65 | 239.60 | 0.001 | 0.00 | 0.238 | 0.016 | 0.414 | 0.025 | 0 .432 | 0 .017 |
| FBTCpos TLE and extra-TLE | 22 | 2003.19 | 309.54 | 0.001 | 0.00 | 0.239 | 0.019 | 0.420 | 0.020 | 0 .433 | 0 .024 |
|  |  | p-value |  | p-value |  | p-value |  | p-value |  | p-value |  |
| FBTCneg TLE *vs* FBTCpos TLE |  | **0.016** |  | 0.240 |  | 0.652 |  | 0.079 |  | 0.295 |  |
| FBTCneg TLE and extra-TLE *vs* FBTCpos TLE and extra-TLE |  | **<0.001** |  | 0.498 |  | 0.831 |  | 0.224 |  | 0.824 |  |

Abbreviations: TLE, temporal lobe epilepsy; MD, mean diffusivity; FA, fractional anisotropy; ODI, orientation dispersion index; NDI, neurite density index.
